# Supplementary material for: Cost-effectiveness analysis of olaparib maintenance therapy for BRCA mutation ovarian cancer in the public sector in Malaysia
Source: PLoS One. 2024 Feb 1;19(2):e0298130. doi: 10.1371/journal.pone.0298130 (PMC10833573; doi:10.1371/journal.pone.0298130)
Supplement: S1 Table — (DOCX) [file pone.0298130.s002.docx]

**S1 Table:** **The rate of transition used in the model**

The current model contains 5 possible transitions, which are summarized below:

• PFS to Death

• PFS to PD1

• PD1 to death

• PD1 to PD2

• PD2 to death

The probabilities assigned to each of the 5 transitions were derived from a series of survival analyses of individual patient-level data in SOLO1, with PD1 being based on the derived PFS endpoint, PD2 on the derived PFS2 endpoint and death being based on OS. Where possible, individual transitions were modelled independently via competing risk equations. The distribution used are summarized below

**Olaparib**

|  | **Distribution** | **Parameter** | **Est** | **Lower 95% CI** | **Upper 95% CI** |
| --- | --- | --- | --- | --- | --- |
| PFS to death | Lognormal | meanlog | 2.5227 | 2.2026 | 2.8428 |
|  |  | sdlog | 1.1816 | 0.96506 | 1.4467 |
| PFS to PD1 | Exponential | rate | 0.043894 | 0.033175 | 0.058077 |
| PD1 to death | Generalised gamma | mu | 2.2985 | 0.33959 | 4.2574 |
|  |  | sigma | 2.269 | 1.3481 | 3.8189 |
|  |  | Q | -3.9412 | -8.4905 | 0.60814 |
| PD1 to PD2 | Lognormal | meanlog | 2.3655 | 2.1514 | 2.5796 |
|  |  | sdlog | 0.90454 | 0.74687 | 1.0955 |
| PD2 to death | Gompertz | shape | 0.058561 | 0.010126 | 0.107 |
|  |  | rate | 0.034141 | 0.018633 | 0.062555 |

**Routine surveillance**

|  | **Distribution** | **Parameter** | **Est** | **Lower 95% CI** | **Upper 95% CI** |
| --- | --- | --- | --- | --- | --- |
| PFS to death | Lognormal | meanlog | 1.5179 | 1.2549 | 1.7808 |
|  |  | sdlog | 0.87903 | 0.70837 | 1.0908 |
| PFS to PD1 | Exponential | rate | 0.12483 | 0.092251 | 0.16891 |
| PD1 to death | Generalised gamma | mu | 1.7514 | 1.421 | 2.0819 |
|  |  | sigma | 0.4594 | 0.032856 | 6.4234 |
|  |  | Q | -30.819 | -107.9 | 46.257 |
| PD1 to PD2 | Weibull | shape | 1.5568 | 1.2314 | 1.968 |
|  |  | scale | 24.634 | 20.39 | 29.76 |
| PD2 to death | Weibull | shape | 0.99973 | 0.62596 | 1.5967 |
|  |  | scale | 41.302 | 20.447 | 83.426 |
