# Supplementary material for: Cost-effectiveness analysis of olaparib maintenance therapy for BRCA mutation ovarian cancer in the public sector in Malaysia
Source: PLoS One. 2024 Feb 1;19(2):e0298130. doi: 10.1371/journal.pone.0298130 (PMC10833573; doi:10.1371/journal.pone.0298130)
Supplement: S2 Table — (DOCX) [file pone.0298130.s003.docx]

**S2 Table: List of subsequent line anticancer regimens used in the model**

| Treatment | Regimen | Source |
| --- | --- | --- |
| Platinum containing regimen | Carboplatin + paclitaxel  Carboplatin + pegylated liposomal doxorubicin | Local clinical expert opinion |
| Non-platinum regimen | Paclitaxel  Pegylated liposomal doxorubicin  Gemcitabine  Topotecan | Local clinical expert opinion |
