# Supplementary material for: Cost-effectiveness analysis of olaparib maintenance therapy for BRCA mutation ovarian cancer in the public sector in Malaysia
Source: PLoS One. 2024 Feb 1;19(2):e0298130. doi: 10.1371/journal.pone.0298130 (PMC10833573; doi:10.1371/journal.pone.0298130)
Supplement: S3 Table — (DOCX) [file pone.0298130.s004.docx]

**S3 Table: Resource utilization for subsequent therapies**

| **Mixture of therapies received** | **% of patient** | **% of regimen** |
| --- | --- | --- |
| **Platinum regimen** | 80% |  |
| Carboplatin + paclitaxel |  | 90% |
| Carboplatin + pegylated liposomal doxorubicin |  | 10% |
|  |  |  |
| **Non-platinum regimen** | 20% |  |
| Paclitaxel |  | 33% |
| Pegylated liposomal doxorubicin |  | 33% |
| Gemcitabine |  | 33% |
| Topotecan |  | 1% |
