# Supplementary material for: Cost-effectiveness analysis of olaparib maintenance therapy for BRCA mutation ovarian cancer in the public sector in Malaysia
Source: PLoS One. 2024 Feb 1;19(2):e0298130. doi: 10.1371/journal.pone.0298130 (PMC10833573; doi:10.1371/journal.pone.0298130)
Supplement: S4 Table — (DOCX) [file pone.0298130.s005.docx]

**S4 Table: Parameters included in the deterministic and probabilistic analysis and their distribution.**

|  | Included as part of PSA | Distribution |
| --- | --- | --- |
| Discounting, Effect | N |  |
| Discounting, Costs | N |  |
| Excess mortality (OC patients with BRCA mutation) | N |  |
| OS: acceleration factor | Y | Normal |
| PF HSUV | Y | Lognormal |
| PF2 HSUV | Y | Lognormal |
| PD HSUV | Y | Lognormal |
| Baseline age | Y | Normal |
| PF health state costs (initial 24m): olaparib | Y | Gamma |
| PF health state costs (>24m): olaparib | Y | Gamma |
| PD1 health state costs: olaparib | Y | Gamma |
| PD2 health state costs: olaparib | Y | Gamma |
| PF health state costs (initial 24m): WW | Y | Gamma |
| PF health state costs (>24m): WW | Y | Gamma |
| PD1 health state costs: WW | Y | Gamma |
| PD2 health state costs: WW | Y | Gamma |
| EOL care (proportion) | Y | Beta |
| EOL care (costs) | Y | Gamma |
| BRCA testing (total costs) | Y | Gamma |
| AE incidence olaparib: Anaemia | Y | Beta |
| AE incidence olaparib: Neutropenia | Y | Beta |
| AE incidence olaparib: Diarrhoea | Y | Beta |
| AE incidence WW: Anaemia | Y | Beta |
| AE incidence WW: Neutropenia | Y | Beta |
| AE incidence WW: Diarrhoea | Y | Beta |
| AE costs: Anaemia | Y | Gamma |
| AE costs: Neutropenia | Y | Gamma |
| AE costs: Diarrhoea | Y | Gamma |
| AE disutilities: Anaemia | Y | Beta |
| AE disutilities: Neutropenia | Y | Beta |
| AE disutilities: Diarrhoea | Y | Beta |
| Treatment duration | N |  |
