# Supplementary material for: Cost-effectiveness analysis of olaparib maintenance therapy for BRCA mutation ovarian cancer in the public sector in Malaysia
Source: PLoS One. 2024 Feb 1;19(2):e0298130. doi: 10.1371/journal.pone.0298130 (PMC10833573; doi:10.1371/journal.pone.0298130)
Supplement: S5 Table — (DOCX) [file pone.0298130.s006.docx]

**S5 Table: Results of deterministic sensitivity analyses conducted in this study**

| **Parameter** | **Lower limit** | **Upper limit** |
| --- | --- | --- |
| Time horizon | 65,227 | 48,206 |
| Discounting, Effects | 35,578 | 69,904 |
| OS: acceleration factor | 76,007 | 44,930 |
| Discounting, Costs | 46,764 | 58,117 |
| PF health state costs (initial 24m): olaparib | 55,172 | 56,529 |
| Age-related utility 55-64 | 52,924 | 55,871 |
| Age-related utility 75+ | 55,579 | 53,188 |
| BRCA testing (total costs) | 53,313 | 55,402 |
| PF health state costs (>24m): olaparib | 54,526 | 55,364 |
| PD2 health state costs: WW | 54,285 | 53,527 |
| PD1 health state costs: WW | 54,287 | 53,551 |
| Age-related utility 65-74 | 55,012 | 53,718 |
| PF health state costs (initial 24m): WW | 54,244 | 53,796 |
| PF HSUV | 54,944 | 53,784 |
| PD2 health state costs: olaparib | 54,394 | 54,775 |
| Excess mortality (OC patients with BRCA mutation) | 54,349 | 54,726 |
| AE costs: Diarrhoea | 54,038 | 54,045 |
| PF health state costs (>24m): WW | 54,329 | 54,038 |
| PD1 health state costs: olaparib | 54,382 | 54,639 |
| PF2 HSUV | 54,081 | 54,637 |
| PD HSUV | 54,139 | 54,577 |
| Age-related utility 45-54 | 54,229 | 54,487 |
| AE duration: Neutropenia | 54,292 | 54,259 |
| AE incidence olaparib: Anaemia | 54,283 | 54,431 |
| AE costs: Anaemia | 54,291 | 54,424 |
| AE duration: Anaemia | 54,316 | 54,296 |
| AE disutilities: Anaemia | 54,335 | 54,335 |
| EOL care (proportion) | 54,371 | 54,344 |
| AE incidence olaparib: Neutropenia | 54,345 | 54,369 |
| AE incidence WW: Neutropenia | 54,365 | 54,350 |
| AE disutilities: Diarrhoea | 54,353 | 54,351 |
| AE incidence WW: Anaemia | 54,363 | 54,352 |
| AE costs: Neutropenia | 54,353 | 54,362 |
| AE disutilities: Neutropenia | 54,355 | 54,355 |
| AE duration: Diarrhoea | 54,356 | 54,359 |
| AE incidence olaparib: Diarrhoea | 54,357 | 54,358 |
